# Metabolic Alternations During Gestation in Dezhou Donkeys and the Link to the Gut Microbiota

**DOI:** 10.3389/fmicb.2022.801976

**Published:** 2022-03-17

**Authors:** Yan Li, Qingshan Ma, Guiqin Liu, Zhenwei Zhang, Yandong Zhan, Mingxia Zhu, Changfa Wang

**Affiliations:** Shandong Engineering Technology Research Center for Efficient Breeding and Ecological Feeding of Black Donkey, College of Agronomy, Liaocheng University, Liaocheng, China

**Keywords:** gestation, donkeys, biochemistry parameters, gut microbiota, association

## Abstract

The maternal intestinal microbial community changes dramatically during pregnancy and plays an important role in animal growth, metabolism, immunity and reproduction. However, our understanding of microbiota compositional dynamics during the whole pregnancy period in donkey is incomplete. This study was carried out to evaluate gut microbiota alterations as well as the correlation with serum biochemical indices, comparing pregnant donkeys to non-pregnant donkeys. A total of 18 pregnant (including EP, early-stage pregnancy; MP, middle-stage pregnancy and LP, late-stage pregnancy) and six non-pregnant (C as a control) donkey blood samples and rectum contents were collected. The results showed that pregnant donkeys had higher microbial richness than non-pregnant donkeys and that the lowest microbial diversity occurred at the EP period. Moreover, the relative abundances of the families Clostridiaceae and Streptococcaceae were significantly higher in the EP group (*p* < 0.05) than that in the C and MP groups, while the relative abundances of the families Lachnospiraceae and Rikenellaceae were significantly lower in the EP group (*p* < 0.05) than that in the C group. The predicted microbial gene functions related to the inflammatory response and apoptosis, such as *Staphylococcus aureus* infection, the RIG-1-like receptor signaling pathway and apoptosis, were mainly enriched in EP. Furthermore, pregnant donkeys had higher glucose levels than non-pregnant donkeys, especially at EP period. EP donkeys had lower triglyceride, total protein and albumin levels but higher malondialdehyde, interleukin 1β, interleukin 6 and tumor necrosis factor-α levels than those in the C and MP groups. Additionally, there were strong correlations between inflammatory cytokine levels and the relative abundances of genera belonging to the Clostridiaceae and Streptococcaceae families. This is the first comparative study performed in donkeys that indicates that pregnancy status (especially in the early pregnancy period) alters the gut microbiota composition, which was correlated with serum biochemical parameters. These results could provide useful information for improving the reproductive management in Dezhou donkeys.

## Introduction

Pregnancy is a unique physiological condition wherein various dramatic physiological changes (including metabolism and immunity) occur compared to the non-pregnant state (Koren et al., 2012; Nair et al., 2017). In regard to physiological changes, biochemical changes in the blood of normal pregnant animals are required, and are important markers reflecting the health status of animals (Shao et al., 2020). In the metabolic state, insulin sensitivity is commonly reduced, especially in the late pregnancy stage (Barbour et al., 2007). Additionally, oxidative stress (Toboła-Wróbel et al., 2020) and immunological challenges (Mor and Cardenas, 2010; Nair et al., 2017) might also occur during the pregnancy period. It has been reported that physiological biochemical changes occur during pregnancy in humans (Dai et al., 2018; Stokkeland et al., 2019), rats (Corvino et al., 2015) and sows (Shao et al., 2020). In the equine ass, several studies have also reported blood biochemical changes during pregnancy, including in horses and donkeys (Vincze et al., 2015; Bonelli et al., 2016; Gloria et al., 2018; Liao Q. et al., 2021), but every species has particular blood biochemical changes related to gestation (Gloria et al., 2018) due to different anatomy and metabolic conditions in different species. Therefore, it is necessary to investigate the species-specific changes in blood biochemical profiles throughout the entire pregnancy period in donkeys. These data could provide a practical basis for the management and diagnosis of gestation in certain donkey breeds.

The gut microbiota has been a topic of great interest due to its important role in body metabolism and immune and physiological functions; thus, it is closely related to host health (Turnbaugh et al., 2006; Kamada et al., 2012; Osbelt et al., 2020). Factors affecting the composition of the gut microbiota and the relationship with the host are of considerable complexity. However, pregnancy, which is a common physiological state, is considered a classical factor that alters the gut microbiota. A significant shift in the gut microbiota during the pregnancy period has been shown (Santacruz et al., 2010; Koren et al., 2012; Huang X. et al., 2019). For example, an increase in the relative abundances of *Actinobacteria* and *Proteobacteria* from the first to the third trimester of pregnancy was reported by Koren et al. (2012). Changes in community structure could result in functional changes, and then affect host health. For instance, adiposity and insulin insensitivity were increased in germ-free mice receiving the intestinal microbiota from women in late pregnancy compared to those in mice inoculated with the gut microbiota of women in early pregnancy (Koren et al., 2012), thus affecting innate immune function and fetal development and health (Gomez de Agüero et al., 2016; Lee et al., 2021) by regulating the incipient microbial biomass and communities of offspring (Perez-Muñoz et al., 2017; Bi et al., 2021). Furthermore, gut microbiota changes may directly influence maternal metabolic alterations related to pregnancy (Koren et al., 2012). Based on association analysis, in humans, it has been indicated that the gut microbiota is correlated with the body weight, weight gain and blood biochemical indices of pregnant women (Santacruz et al., 2010). Regarding the relationships between the gut microbiota and blood biochemical parameters, an increasing number of studies have been carried out. In laboratory animals, Hua et al. (2018) showed that the relative abundances of *Romboutsia* and *Phascolarctobacterium* were positively associated with the serum triglyceride (TG) level in a high-fat diet-fed rat model. In addition, it has been reported that blood urea nitrogen levels were negatively correlated with *Ruminococcaceae* in a sow model (Shao et al., 2020). However, in breeding donkeys, information regarding how the gut microbiota varies throughout pregnancy is limited. Likewise, little information is known about whether and how the gut microbiota contributes to serum biochemical changes during the normal pregnancy period compared with the non-pregnancy state. To address this deficiency, we hypothesized that the mother donkey exhibits blood physiology changes and dramatic changes in the gut microbiota during the pregnancy period and that blood metabolic disorders and immune injury are caused by microbial composition changes.

The aim of the present study was to evaluate the dynamic changes in the gut microbiota in donkeys during the pregnancy period. Biomarker monitoring was conducted to assess metabolic functional and health status across different pregnancy stages. Moreover, the association between changes in the levels of biomarkers and gut bacteria was also identified. Data obtained from this study will provide useful information for improving the reproductive management in Dezhou donkeys.

## Materials and Methods

### Animal Selection, Husbandry and Sample Collection

A total of 24 healthy donkeys ranging between 3 and 5 years of age were selected for this study. According to their pregnancy stages, the donkeys were divided into four groups: the non-pregnant (C, as a control), early stage of pregnancy (EP, between 1 and 3 months), middle stage of pregnancy (MP, between 6 and 9 months) and late stage of pregnancy (LP, 1 month before parturition) groups. Each group consisted of six animals. All donkeys were raised under the same conditions at a Dezhou donkey original breeding farm authorized by Shandong Province (Dezhou city, Shandong, China). Female donkeys were fed a commercial concentrate diet (Hekangyuan Group Co., Ltd., Shandong, China). Non-pregnant and pregnant donkeys were administered twice daily (08:00 and 16:00) at 0.25 and 1.5% of their body weight, respectively. Wheat straw (ratio of 60:40) and water were provided *ad libitum* throughout the rearing period. Additionally, none of the donkeys had received antibiotics for at least 3 months before sampling. The animal care protocol in this study followed commercial management practice and was approved by the Animal Welfare Committee of Liaocheng University.

All the samples were collected between 9 and 11 am on the same day. Blood samples were collected in tubes (5 mL) from a jugular vein before feeding. Serum samples were obtained after centrifugation at 3,000 × *g* for 10 min at 4°C and then snap frozen in liquid nitrogen. Fecal samples were obtained from donkey rectum content, transferred to separate sterilized 5 mL tubes, and then stored immediately in liquid nitrogen for DNA extraction. Then, all frozen samples stored in dry ice were transported to the laboratory and stored at −80°C for further analysis.

### Physiological Analyses of the Serum

The analysis of glucose (GLU), TG, total protein (TP), albumin (ALB), total cholesterol (TC), total bilirubin (T-BIL), aspartate aminotransferase (AST), alanine aminotransferase (ALT), alkaline phosphatase (ALP), gamma-glutamyl transferase (γ-GT), and creatine kinase (CK) levels in serum was carried out using a BS-420 automated chemical analyzer (Mindray, China). Furthermore, malondialdehyde (MDA), total superoxidase dismutase (T-SOD), and glutathione peroxidase (GSH-Px) levels and total antioxidant capacity (T-AOC), as biomarkers of oxidation and antioxidation states, were determined with a chemistry analyzer (Commercial Kit, Nanjing Jiancheng Bioengineering Institute, China) in accordance with the manufacturer’s recommended procedures. In addition, the interleukin 1β (IL-1β), interleukin 6 (IL-6) and tumor necrosis factor-α (TNF-α) levels, as biomarkers of inflammation, were measured with the corresponding equine-specific ELISA kits (CUSABIO Biotech, Wuhan, China) according to the manufacturer’s instructions.

### DNA Extraction and PCR Amplification

Samples were obtained from the rectum content of donkeys in different gestation periods (*n* = 6) and then used for bacterial composition analysis. Genomic DNA was isolated using an E.Z.N.A.^®^ Soil DNA Kit (Omega Bio-tek, Norcross, GA, United States) according to the manufacturer’s instructions. DNA yield and quality were tested with a NanoDrop2000 (Thermo Scientific, Wilmington, United States). The V3-V4 region of the bacterial 16S rRNA gene was amplified by a thermocycler PCR system (Gene Amp 9700, ABI, United States) using the primers 338F (5′-ACTCCTACGGGAGGCAGCAG-3′) and 806R (5′-GGACTACHVGGGTWTCTAAT-3′). PCRs were performed in triplicate in a 20 μL mixture containing 4 μL of 5 × FastPfu Buffer, 2 μL of 2.5 mM dNTPs, 0.8 μL of each primer (5 μM), 0.4 μL of FastPfu Polymerase, 0.2 μL of BSA, and 10 ng of template DNA. The PCR conditions were as follows: 95°C for 3 min to allow DNA denaturation, with amplification lasting 27 cycles (95°C for 30 s, 55°C for 30 s and 72°C for 45 s) and a final extension period lasting for 10 min at 72°C.

### Illumina MiSeq Sequencing and Bioinformatic Analysis

The purified amplicons were pooled in equimolar amounts and paired-end sequenced on an Illumina MiSeq platform (Illumina, San Diego, United States) according to standard protocols by Majorbio Bio-Pharm Technology Co., Ltd. (Shanghai, China). Raw fastq files were quality filtered by Trimmomatic and merged by FLASH (Magoè and Salzberg, 2011). Operational taxonomic units (OTUs) were clustered with a 97% similarity cutoff using UPARSE (version 7.1) with a novel “greedy” algorithm that simultaneously performs chimera filtering and OTU clustering (Edgar, 2013). The taxonomy of each 16S rRNA gene sequence was analyzed by the RDP Classifier algorithm against the Silva (SSU123) 16S rRNA database using a confidence threshold of 70%. To minimize the effects of sequencing depth on alpha and beta diversity measure, the number of reads from each sample was rarefied to 24,581. OTUs diversity and richness estimators were determined on the basis of the Shannon index and Simpson index (diversity) and the abundance-based coverage estimator (ACE) and bias-corrected Chao estimator (Chao 1) (richness) using the MOTHUR (version 1.30.2) program. A Venn diagram shows the number of OTUs shared among the groups. β-diversity was calculated by measuring the Bray-Curtis distance using QIIME (version 1.9.1) software, and visualized using principal coordinate analysis (PCoA). Taxonomic community composition was analyzed through the visualization of the data sets of the relative abundances in different samples. By performing linear discriminant analysis coupled with effect size (LEfSe), we identified the most differentially abundant taxa between different groups (LDA > 3.5). Finally, microbial functions were predicted using phylogenetic investigation of communities by reconstruction of unobserved states (PICRUSt) (Langille et al., 2013). Additionally, raw data of high throughput sequencing have been deposited to the NCBI Sequence Read Archive with accession no. PRJNA784020.

### Bacterial DNA Quantitative PCR

16S rRNA gene copies of the phyla Firmicutes and Bacteroidetes were determined in the rectum contents of donkeys by real-time quantitative PCR (qPCR) as previously described (Li et al., 2018). In detail, the genes were quantified with a standard curve for gene copy number by cloning specific primer sequences into pMD18-T plasmids. Standard curves were constructed from 10^8^ to 10^0^ (10-fold serial dilutions) copies of amplified bacterial 16S rRNA genes from reference strains. The primer information for specific bacteria is listed in Supplementary Table 1. The qPCR assay was performed with an ABI7300 PCR Detection System (Applied Biosystems) with a ChamQ SYBR Color qPCR Master Mix (2X) Kit (Vazyme Biotech Co., Ltd., Nanjing, China). The PCR results were expressed as 16S rRNA gene copies per gram (copies g^–1^) of wet fecal sample. All measurements were performed in duplicates.

### Statistical Analyses

Data on the serum biochemical parameters and qPCR results (16S rRNA gene copies of the phyla Firmicutes and Bacteroidetes) were subjected to one-way ANOVA followed by Duncan multiple comparison using the SPSS statistical software package (version 22). The variability of the results is expressed as the mean ± standard error. Means were considered significantly different at *p* < 0.05. The results were performed using the GraphPad Prism 6.0 version.

Gut bacterial data were analyzed on the online platform of Majorbio Cloud Platform^1^. We used a Wilcoxon rank-sum test to determine the variance of α diversity index of gut microbiota. PCoA and NMDS plots based on the Bray-Curtis distance were used to visualize differences in bacterial community composition among samples. R 4.0.3 was used perform the differences in PICRUSt between groups. Finally, connections between biochemical indices and microbial abundances at the family and genus levels were evaluated by Spearman’s correlation coefficients based on the heatmap analysis and visualized using R (version 3.3.1), and *p* < 0.05 were considered to be significantly correlated.

## Results

### Serum Biochemical Parameters in Donkeys

The EP group had the highest GLU content, while the MP and LP groups showed a higher trend than the C group (Table 1). The EP group showed significantly lower levels of TG, TP, ALB and T-BIL in serum than the other groups (*p* < 0.05; Table 1). The TC level during pregnancy (EP, MP and LP) was significantly lower than that in non-pregnancy (C group), which was opposite to the change in AST activity. The activity of serum ALP was significantly highest in the LP group, while there was a higher trend in the C and EP groups than in the MP group (Table 1). The activity of serum CK was significantly higher in the EP and LP groups than in the C and MP groups (*p* < 0.05; Table 1). Moreover, the activities of ALT and γ-GT in serum presented no differences among the four groups (Table 1).

**TABLE 1:** Serum biochemical parameters in Dezhou donkeys during different gestation stages.

Index	C	EP	MP	LP
GLU (mmol/L)	3.95 ± 0.20^b^	4.36 ± 0.05^a^	4.20 ± 0.06^ab^	4.10 ± 0.07^ab^
TG (mmol/L)	0.74 ± 0.07^a^	0.35 ± 0.05^b^	0.85 ± 0.06^a^	0.70 ± 0.13^a^
TP (g/L)	63.09 ± 1.48^a^	55.99 ± 1.52^b^	62.28 ± 1.70^a^	61.48 ± 1.77^a^
ALB (g/L)	24.01 ± 0.31^a^	19.48 ± 0.69^b^	22.98 ± 0.40^a^	23.30 ± 0.57^a^
TC (mmol/L)	1.83 ± 0.07^a^	1.43 ± 0.08^b^	1.39 ± 0.01^b^	1.52 ± 0.06^b^
T-BIL (μmol/L)	10.12 ± 0.69^a^	7.60 ± 0.25^b^	8.96 ± 0.31^a^	9.69 ± 0.13^a^
AST (U/L)	184.01 ± 7.83^b^	258.30 ± 5.91^a^	270.88 ± 10.98^a^	259.44 ± 13.61^a^
ALT (U/L)	8.50 ± 0.71	10.10 ± 0.56	9.03 ± 0.71	10.12 ± 1.03
ALP (U/L)	14.68 ± 0.34^ab^	15.10 ± 2.87^ab^	10.66 ± 1.41^b^	17.65 ± 1.57^a^
γ-GT (U/L)	19.57 ± 1.31	21.60 ± 1.56	19.02 ± 0.74	20.48 ± 1.09
CK (U/L)	148.29 ± 9.17^c^	227.60 ± 14.36^b^	154.16 ± 11.11^c^	339.98 ± 37.28^a^

*GLU, glucose; TG, triglyceride; TP, total protein; ALB, albumin; TC, total cholesterol; T-BIL, total bilirubin; AST, aspartate aminotransferase; ALT, alanine aminotransferase; ALP, alkaline phosphatase; γ-GT, gamma-glutamyl transferase; CK, creatine kinase. C, non-pregnancy as a control; EP, early-stage pregnancy; MP, middle-stage pregnancy; LP, late-stage pregnancy.*

*^a,b^Means within the same line with different superscript are significantly different, p < 0.05.*

**FIGURE 1:**
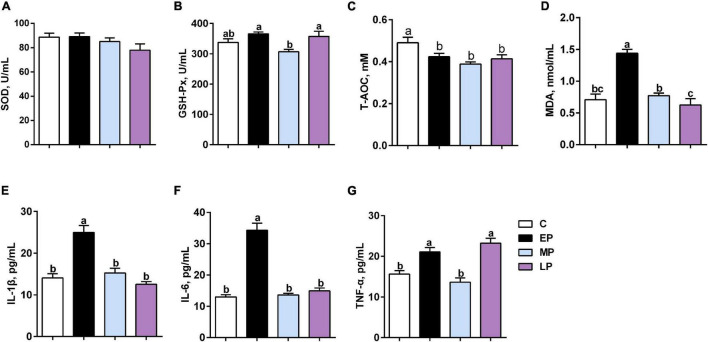
Serum oxidative status and inflammation in Dezhou donkeys during different gestation stages. **(A)** SOD (superoxide dismutase); **(B)** GSH-P_*X*_ (glutathione peroxidase); **(C)** T-AOC (total antioxidant capacity); **(D)** MDA (malondialdehyde); **(E)** IL-1β (interleukin-1 beta); **(F)** IL6 (interleukin 6); and **(G)** TNF-α (tumor necrosis factor alpha). C, non-pregnancy as a control; EP, early-stage pregnancy; MP, middle-stage pregnancy; LP, late-stage pregnancy. Data indicate means ± SEM (*n* = 6). *^a,b,c^*Means with different letters are significantly different, *p* < 0.05.

There was no change in SOD activity among the four groups (Figure 1A). GSH-P_*X*_ activity in serum was significantly higher in the EP and LP groups than in the MP group (*p* < 0.05; Figure 1B) but had a higher trend than that in the C group. However, the T-AOC levels during pregnancy (EP, MP and LP) were significantly lower than those during non-pregnancy (C group; *p* < 0.05; Figure 1C). In addition, the level of MDA in serum was significantly highest in the EP group, whereas the trend was lower in the LP group than in the C group (*p* < 0.05; Figure 1D).

The levels of the proinflammatory cytokines IL-6 and IL-1β were highest in EP (*p* < 0.05; Figures 1E,F). Moreover, the level of TNF-α was significantly higher in the EP and LP groups than in the C and MP groups (*p* < 0.05; Figure 1G). Therefore, our data suggest that metabolic disorders and low-grade inflammation might exist in donkeys during the gestation period, especially at the early stage (in the EP group).

### Diversity Changes in the Gut Microbiota

**FIGURE 2:**
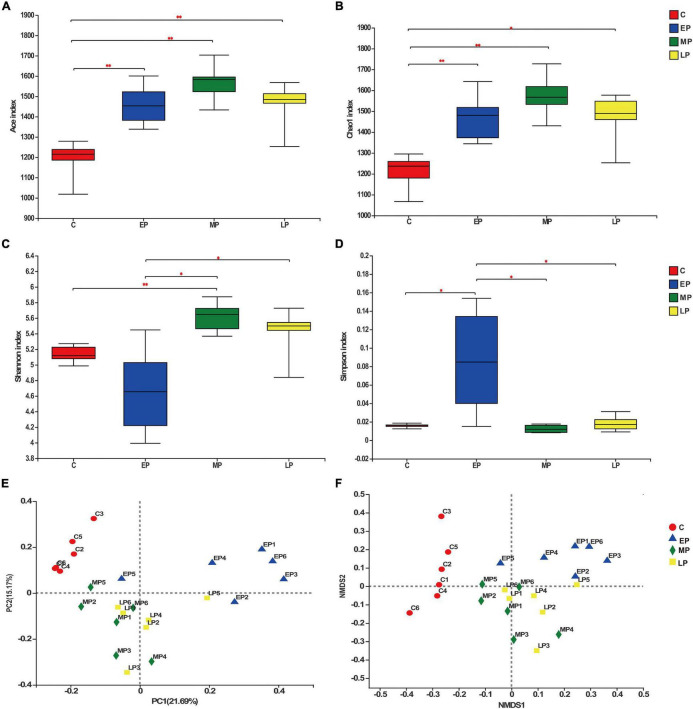
The biodiversity of the gut microbiota is significantly changed during different gestation stages. Richness determined by the Ace **(A)** and Chao 1 indices **(B)** and α diversity determined by the Shannon **(C)** and Simpson indices **(D)**. Principal coordinate analysis [PCoA; **(E)**] and non-metric multidimensional scaling [NMDS; **(F)**] analyses based on Bray-Curtis distance showing the differences in the gut microbiota in different gestation stages. C, non-pregnancy as a control; EP, early-stage pregnancy; MP, middle-stage pregnancy; LP, late-stage pregnancy. Data indicate means ± SEM (*n* = 6), *p < 0.05, ^**^p < 0.01.

To evaluate the effect of pregnancy status on the donkey fecal microbiota, we collected 24 rectum content samples from donkeys in different pregnancy periods and non-pregnancy and analyzed the bacterial community structure by 16S rRNA high throughput sequencing. After quality control, a total of 1,250,751 high-quality sequences were obtained from 24 samples with an average of 52,114 ± 6007 sequences per sample. In addition, 2,499 OTUs were obtained based on the 97% sequence similarity level, 1,399 of which existed in all groups and were thus defined as core OTUs (Supplementary Figure 1). The core OTUs comprised 56.0% of the total OTUs, whereas 65, 44, 52, and 64 OTUs were uniquely identified in the C, EP, MP, and LP groups, respectively (Supplementary Figure 1). Diversity differences between groups were assessed. The abundance and α-diversity of the microorganisms among the pregnancy periods were observed. The observed ACE and Chao 1 indices were lowest in the C group (Figures 2A,B). No remarkable change was noted in the observed Chao 1 and ACE indices among the EP, MP, and LP donkeys. The Shannon index in the EP group was lower than that in the MP and LP groups, and there was no change between the C and EP groups (Figure 2C). Moreover, the Simpson index in the EP group was higher than that in the C, MP, or LP groups (Figure 2D). The dramatic changes in the four indices indicated that the community richness and diversity of the four groups were varied. In addition, through PCoA based on Bray–Curtis dissimilarity, we found that the fecal microbiota of donkeys was obviously segregated in the non-pregnant group (control) and pregnant groups, especially between in the control and EP groups, but was less dispersed in the MP and LP groups (Figure 2E). Moreover, the results of the non-metric multidimensional scaling (NMDS) revealed a similar change pattern (Figure 2F). Taken together, the fecal bacterial components and diversity of donkeys is profoundly altered during pregnancy.

### Composition Changes of the Fecal Microbiota

**FIGURE 3:**
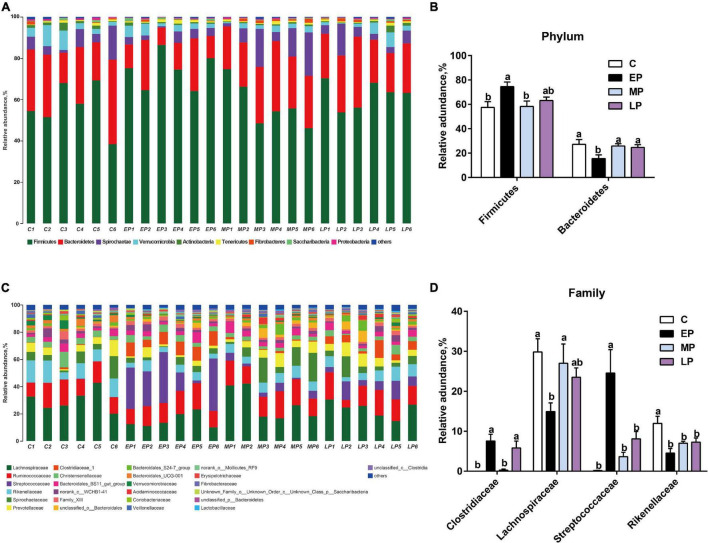
The distinct bacterial composition of the gut microbiota in Dezhou donkeys during different gestation stages. **(A)** The gut bacterial composition at the phylum level in donkeys during different gestation stages. **(B)** The relative abundance of the phylum Firmicutes and Bacteroidetes in donkeys during different gestation stages. **(C)** The gut bacterial composition at the family level in donkeys during different gestation stages. **(D)** The relative abundances of the families Clostridiaceae, Lachnospiraceae, Streptococcaceae, and Rikenellaceae in donkeys during different gestation stages. C, non-pregnancy as a control; EP, early-stage pregnancy; MP, middle-stage pregnancy; LP, late-stage pregnancy. Data indicate means ± SEM (*n* = 6), *^a,b^*Means with different letters are significantly different, *p* < 0.05.

**FIGURE 4:**
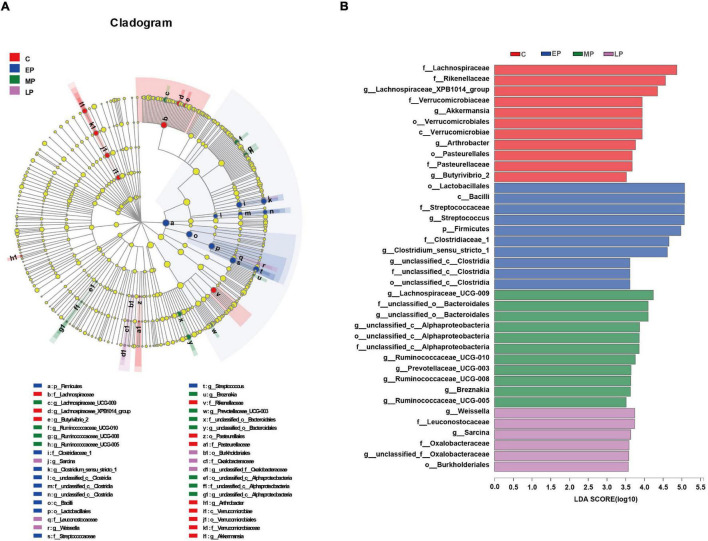
LEfSe analysis of the gut microbial composition of Dezhou donkeys during different gestation stages. **(A)** Cladogram obtained from the LEfSe method, indicating the phylogenetic distribution of the gut microbiota. **(B)** Histogram of the LDA scores, showing the most differentially abundant taxa among different gestation stages (LDA score > 3.5, *n* = 6). C, non-pregnancy as a control; EP, early-stage pregnancy; MP, middle-stage pregnancy; LP, late-stage pregnancy.

We then further studied the changes in fecal community phylotypes among the four groups. The relative abundance (%) of the gut microbiota in the four groups at the phylum and family levels is shown in Figure 3. As expected, we found that the dominant bacterial phyla in the feces in all donkeys were Firmicutes and Bacteroidetes (Figures 3A,B), which accounted for more than 80% of the relative abundance. After comparing the differences among the four groups, it was found that the relative abundance of Firmicutes in the EP group was significantly higher than that in the C group (*p* < 0.05; Figure 3B) but had a higher tendency at the LP stage. However, the relative abundance of the phylum Bacteroidetes was the lowest at EP stage (*p* < 0.05; Figure 3B). Furthermore, we quantified their abundances by using qPCR and observed a similar result (Supplementary Figure 2). The increased abundance of Firmicutes in the EP group was mainly attributed to the enrichment of *Clostridiaceae* and *Streptococcaceae*, whereas the decreased abundance of Bacteroidetes was primarily due to the depletion of Rikenellaceae (Figures 3B,D). The family Lachnospiraceae also belongs to the phylum Firmicutes, but its relative abundance at the EP stage was significantly lower than that in the C and MP groups (*p* < 0.05; Figure 3D). At the genus level, the abundances of *Clostridium_sensu_stricto_*1 and *Streptococcus* in the EP group were the highest (*p* < 0.05; Supplementary Figure 3). Moreover, LEfSe confirmed the above results (Figure 4). Overall, these results indicate that the gut microbiota composition of donkeys is profoundly altered during the pregnancy period, especially at the EP stage.

### Metabolic Functional Changes of the Fecal Microbiota

**FIGURE 5:**
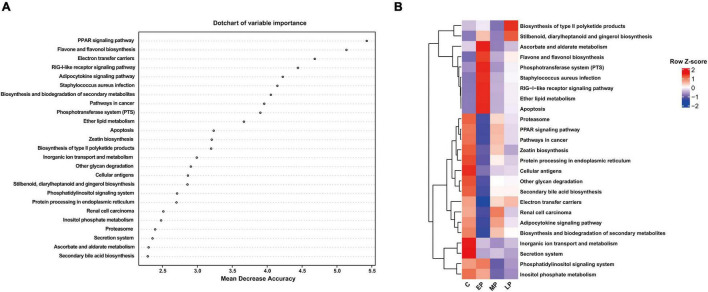
Important bacterial functions identified using random forest analysis. **(A)** The top 25 important bacterial functions according to random forest analysis. **(B)** The distribution of the important bacterial functions in different groups. C, non-pregnancy as a control; EP, early-stage pregnancy; MP, middle-stage pregnancy; LP, late-stage pregnancy.

Based on the significant changes in the bacterial composition of the fecal microbiota, we then analyzed metabolic functional differences. PICRUSt was used to predict the metabolic function of the microbiome based on the results from the 16S rRNA gene sequencing at KEGG taxonomy level 3. We observed the important bacterial functions (top 25) identified by random forest analysis and a heatmap based on the KEGG data (Figures 5A,B). Sixteen pathways (e.g., the proteasome, PPAR signaling, cancer, zeatin biosynthesis, protein processing in endoplasmic reticulum, cellular antigens, other glycan degradation, secondary bile acid biosynthesis, electron transfer carriers, renal cell carcinoma, and adipocytokine signaling pathways) were significantly more abundant in the C group. Ten pathways (e.g., the *Staphylococcus aureus* infection, RIG-1-like receptor signaling pathway, ether lipid metabolism and apoptosis pathways) were enriched in the EP group. Eight pathways (e.g., the proteasome, PPAR signaling, cancer, and zeatin biosynthesis pathways) were more abundant in the MP group, while two other pathways (e.g., type II polyketide product biosynthesis and stilbenoid, diarylheptanoid and gingerol biosynthesis) were more abundant in the LP group. Altogether, these data suggest that pregnancy alters the metabolic functions of the fecal microbiota and therefore deserves further exploration. Additionally, the fecal microbial functions were clearly separated between the C and EP groups and were similar between the MP and LP groups, which was consistent with the β-diversity analysis (Figures 2E,F).

### Correlation of the Gut Microbiota With Biochemical Parameters

**FIGURE 6:**
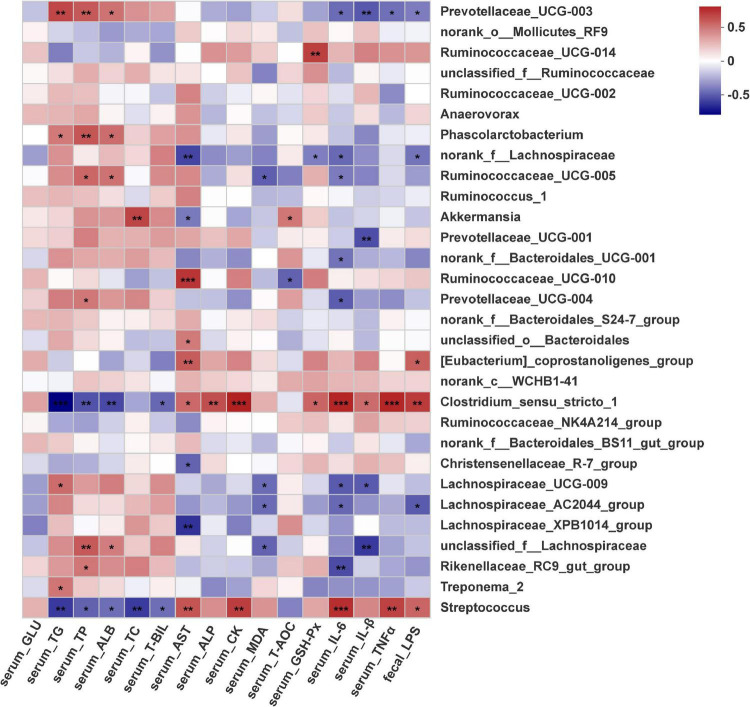
Correlation plot showing Spearman’s correlations among serum metabolism, oxidative status and inflammation, fecal LPS and core genera (top 30). *p < 0.05, **p < 0.01, ***p < 0.001.

The gut microbiota plays a critical role in host metabolism and immune function. Thus, Spearman correlation analysis was performed to evaluate potential associations between the changes in the gut microbiota at the family and genus levels and biochemical parameters included among the sixteen significantly altered profiles in the four groups, and these correlations are represented in heatmaps (Supplementary Figure 4 and Figure 6). Interestingly, the Clostridiaceae_1 and Streptococcaceae families displayed a very similar pattern of correlations with most serum biochemistry parameters, and these correlative patterns were in contrast to the patterns observed for the Lachnospiraceae and Rikenellaceae families (Supplementary Figure 4). At the genus level (Figure 6), two genera were positively associated with serum proinflammatory cytokines (IL-6, IL-1β and TNF-α), implying a positive correlation with the inflammatory response. Conversely, ten genera were negatively correlated with serum IL-6, IL-1β and TNF-α levels, suggesting that these genera were negatively correlated with inflammation status. However, nine genera were positively associated with serum lipids and protein metabolism, and two genera were negatively associated with TG, TC, TP and ALB. Notably, genera abundantly enriched in the EP groups included *Streptococcus* and *Clostridiaceae_sensu_stricto*_1, which were significantly, positively correlated with increased serum IL-6, IL-1β and TNF-α levels but negatively correlated with decreased serum lipid content and protein content. The abundances of *Rikenellaceae_RC9_gut_group*, *unclassifized_f_lachnospiraceae*, *Lachnospiraceae_UCG_009*, *Prevotellaceae_UCG_003*, *Ruminococcaceae_UCG_005* and *Prevotellaceae_UCG_003* were significantly (*p* < 0.05) negatively associated with serum IL-6, IL-1β and TNF-α levels but positively associated with serum lipid content and protein content. The abundance of *Akkermansia* was significantly, positively associated with serum T-AOC and TC contents but negatively (*p* < 0.05) associated with serum AST content. In addition, the abundances of *Streptococcus*, *Clostridiaceae_sensu_stricto_1* and *[Eubacterium]_coprostanoligenes_group* were positively associated with fecal LPS concentration. However, the abundances of *Lachnospiraceae_AC2044 _group*, *norank_f_Lachnospiraceae* and *Prevotellaceae_UCG_003* were negatively correlated with fecal LPS concentration, and serum IL-6 and MDA levels.

## Discussion

Pregnancy is a period of dramatic shift and adaptation as a mother. It is believed that the gut microbiota plays a fundamental role in responding and adapting to the host environment, and then supports host health and normal reproduction. In addition, serum physiological biochemical parameters are also considered as useful biomarkers for monitoring physiological responses and host health. Remarkable changes occur in the gut microbiota during the pregnancy period in humans, rats and several domestic animals. However, little is known about whether the donkey exhibits similar changes in the gut microbiota and serum biochemical parameters during the whole pregnancy period. Therefore, we first investigated the difference of gut microbiota composition as well as the association with serum biochemical indices in different stages of pregnant donkeys. Our results suggest the dramatic changes in fecal microbiota diversity and composition as well as serum biochemical parameters in donkeys during the pregnancy period, especially at the early stage of pregnancy.

In this study, pregnant donkeys (EP, MP and LP) exhibited increased gut microbial richness. However, with regard to evenness, we observed that the Shannon index was lower and the Simpson index was higher in the EP group than in the other three groups, indicating that the additional richness was not evenly distributed during the pregnancy period, especially in the EP group. This finding is similar to the result reported by Koren et al. (2012) who noted that the diversity of the gut microbial community decreased at 1 month postpartum (Koren et al., 2012). This similarity may be because donkeys exhibit foal heat breeding, which results in the simultaneous existence of pregnancy and breastfeeding in the early stages of pregnancy. Moreover, the β diversity results demonstrated significant differences in the microbial composition between the non-pregnant (C) and pregnant donkeys (EP, MP and LP), while the MP and LP groups mostly clustered together. The change indicated that the composition of gut microbes was prone to modulation by the early stage of pregnancy and then gradually stabilized. The greatest effects on α and β diversity were exhibited in the early stage of pregnancy compared to those during the other stages (MP and LP), which is consistent with the change in serum biochemical indices in the study subjects.

In addition to gut microbial diversity, the microbial community composition in donkeys also shifts during different stages of pregnancy. In this study, the dominant phyla found in the donkey fecal microbiota in all groups were Firmicutes and Bacteroidetes (Figure 3A), consistent with previous studies showing that the most abundant phyla were Firmicutes and Bacteroidetes with respect to the breeding stages (Kong et al., 2016; Cheng et al., 2018; Shao et al., 2020). In the equine gut, Firmicutes generally displays the highest relative abundance, followed by Bacteroidetes (Venable et al., 2016; Hashimoto-Hill and Alenghat, 2021), which is similar to our study. Furthermore, we found that the relative abundance of the phylum Firmicutes was highest in EP, whereas Bacteroidetes showed the opposite result. The LP group had a higher trend in the relative abundance of the phylum Firmicutes than the C and MP groups. We next quantified their abundances by using qPCR. A previous study showed that an increase in Firmicutes abundance is considered to support the fetal growth by enhancing energy metabolism (Cheng et al., 2018). In addition, we found lower serum concentrations of TG, TP, and ALB in the EP group (lactating donkeys) than in the other groups (C, MP and LP), which is similar to previous results in Chinese Liaoxi donkeys (Liao Q. et al., 2021). Based on its foal heat breeding character, as we mentioned, these results could be explained by the increase in energy metabolism, nutrition (such as lipid and protein) transfer for milk yield in the mammary glands and fetal development during EP. Similarly, we also found an increase in inflammation and a decrease in total antioxidant ability in EP and LP. However, the inflammatory response in the MP group did not change compare to that in the C group. These findings regarding inflammation were consistent with an earlier report by Mor and Cardenas (2010). The first trimester and third trimester of pregnancy are associated with an inflammatory response, which is necessary for blastocyst implantation and labor, respectively. The second trimester is commonly characterized by an anti-inflammatory status, which is required for fetal growth (Mor and Cardenas, 2010). Accounting for this information, we should consider adopting different management modes according to the different aspects of metabolism, immunity and the microflora during pregnancy, especially in EP in donkeys. On the other hand, the shift in the gut microbiota might be closely linked with physiological changes.

Remarkably, bacteria in the phylum Firmicutes were EP biomarkers (Figure 4B). Likewise, the genera *Streptococcus* (order Lactobacillales) and *Clostridium_sensu_stricto_1* (order Clostridia) were also enriched in EP. A previous study showed that compared with prepartum mares, postpartum mares had an increased relative abundance of the Firmicutes phylum (specifically family Streptocococcaceae) and a corresponding decrease in the relative abundance of the family Lachnospiraceae (also in the Firmicutes phylum) (Weese et al., 2015). Generally, *Clostridium sensu stricto* and *Streptococcus* commonly considered two opportunistic pathogens of the animal intestine (Milinovich et al., 2008; Boyle et al., 2018; Liang et al., 2018; Li et al., 2021). For instance, enrichment of *Streptococcus* spp. has been linked with a disturbance in the microbial community in oligofructose-induced laminitis (Milinovich et al., 2008). Notably, we also observed that the serum proinflammatory cytokine (IL-6, IL-1β and TNF-α) concentrations in donkeys were increased in EP, and Spearman correlation analysis showed that the relative abundances of *Streptococcus* and *Clostridium_sensu_stricto_1* were positively correlated with the levels of biomarkers of systemic low-grade inflammation. In contrast, recent evidence suggests that the decreased abundance of *Clostridium_sensu_stricto_1* in intrauterine growth restricted (IUGR) piglets is negatively correlated with plasma proinflammatory cytokine (IL-1β, TNF-α, and IFN-γ) levels (Huang S. et al., 2019). This difference might be explained by the fact that *Clostridium* has been identified as a highly diverse genus, that contains both potential pathogens and beneficial species (Venable et al., 2016). In addition, *Streptococcus*, one of the major genera in the horse gut (Venable et al., 2016), was the predominant genus in the feces of donkeys at EP in this study. *Streptococcus*, a starch-utilizing bacterium observed in the horse gastrointestinal tract (Goodson et al., 1988), is also considered beneficial to animal health due to its complex interaction with the host. However, although these changes occur in donkeys in EP, the implication of causality and their interplay between enrichment of *Streptococcus* and *Clostridium_sensu_stricto_1* and low-grade inflammation in donkeys remain to be further confirmed. In addition to potential opportunistic pathogens, the abundances of some beneficial bacteria were also influenced in EP. For instance, Lachnospiraceae, which has been associated with maintaining gut health (Vojinovic et al., 2019) and strongly negatively correlated with intestinal inflammation (Zhao et al., 2017), was less abundant on average in EP. Furthermore, Spearman correlation analysis showed that the abundances of *Lachnospiraceae_XPB1014_group*, *Lachnospiraceae_AC_2044* and *Lachnospiraceae_UCG-009* (family Lachnospiraceae) were negatively correlated with the increased levels of serum biomarkers of inflammation of in donkeys in EP. Lachnospiraceae and Ruminococcaceae were mainly enriched in MP. Genera from the families Lachnospiraceae and Ruminococcaceae exhibit anti-inflammatory functions including *Ruminococcaceae_UCG-005*, and are also reported to be involved in the production of short-chain fatty acids (SCFAs) (Liu et al., 2019; Vojinovic et al., 2019; Li et al., 2021; Liao R. et al., 2021), which are essential for the regulation of intestinal microbiota balance and the maintenance of intestinal epithelium integrity (Tan et al., 2014; Kelly et al., 2015). Moreover, Ruminococcaceae bacteria have the ability to degrade cellulose and starch, which is closely related to feed efficiency in herbivorous animals (Zhao et al., 2018). The main biomarkers of LP belonged to the Proteobacteria phylum and Burkholderiales order. It has been reported that the enrichment of Proteobacteria is also closely related to gut dysfunction (Litvak et al., 2017), although Proteobacteria play a minor role in maintaining gut balance (Eckburg et al., 2005). This finding is similar to the results of another study showing that the abundance of the order Burkholderiales (phylum Proteobacteria) in LP of Meishan sows was higher than that in EP and confirmed that elevated Burkholderiales abundance also contributes to inflammation (Xue et al., 2017). We also observed that the level of the proinflammatory cytokine TNF-α was increased in LP. Similarly, a significant relative increase in Proteobacteria abundance was found in women during LP, which caused inflammatory responses in germ-free mice (Koren et al., 2012). Although we found the pregnant donkeys might undergo metabolic disturbances including in fat and protein metabolism and low-grade inflammation, during pregnancy, especially in EP, any interpretation is limited due to small sample number.

PICRUSt was used to observe the metabolic functional changes among the four groups. Microbial gene functions such as *Staphylococcus aureus* infection, the RIG-1-like receptor signaling pathway and apoptosis were enriched in EP, and were related to the inflammatory response and apoptosis. The enrichment of the *Sraphylococcus aureus* infection pathway is related to aggravated intestinal inflammation and hence leads to impaired intestinal barrier function (Hauck and Ohlsen, 2006). The RIG-I-like receptor is an intracellular pattern recognition receptor that specifically recognizes viruses (Yoneyama and Fujita, 2009), which indicates the risk of viral or bacterial infection at EP. Our microbial function results imply that changes in metabolic disorders and the inflammatory response are closely related to the shifts in the gut microbiota at EP. However, functions linked with anti-inflammatory pathways, such as the PPAR pathway and zeatin biosynthesis, were significantly enriched in the gut microbiome in MP. In addition, the predicted pathways involved in the type II polyketide product biosynthesis and stilbenoid, diarylheptanoid and gingerol biosynthesis were enriched in LP. Polyketide-synthesizing bacteria are strongly associated with chronic intestinal inflammation (Arthur et al., 2012). Moreover, in post-weaning diarrhea pigs, the increased abundance of the type II polyketide product biosynthesis pathway might be responsible for the increased inflammatory response (Dou et al., 2017; Hashimoto-Hill and Alenghat, 2021). However, stilbenoid diarylheptanoid and gingerol, secondary metabolites of plants, have been reported to have anti-inflammatory or anticancer activities (Park et al., 1998; Yadav et al., 2003). For example, the stilbenoid, diarylheptanoid, and gingerol biosynthesis pathway has been found to be enriched in high-body-weight rabbits (Zeng et al., 2015). These data indicated that it might be a compensatory mechanism to ameliorate microbial dysbiosis by regulating these microbial processes.

In summary, the present study suggests that pregnant donkeys might undergo metabolic disturbances including in fat and protein metabolism and low-grade inflammation, during pregnancy, especially in EP. The gut microbiota of donkeys changes dramatically throughout pregnancy. The representative changes included an increase in bacterial richness throughout pregnancy, a decrease in bacterial diversity and the relative abundances of Lachnospiraceae and Rikenellaceae in EP and LP, and an increase in the relative abundances of Clostridiaceae and Streptococcaceae in EP. Functional prediction was also influenced by the different pregnancy stages. In addition, the metabolic disturbance in the serum of pregnant donkeys, at least in part, is attributable to the shift in the gut microbiota, especially EP. Therefore, this study provides systematic data on the gut microbiota shift and host metabolism of donkeys throughout pregnancy. However, a major limitation of this study was that samples were obtained from different animals in different pregnancy stages, which might result in variability derived from the different individuals. Another limitation of this study is small sample size. Further research is needed to monitor the shift in microbes in the same individuals while at the same time increasing the sample sizes, and then elucidate the mechanisms involved in cross-talk between the intestinal microbiota or their metabolites and host metabolism and its role in host health.

## Data Availability Statement

The datasets presented in this study can be found in online repositories. The names of the repository/repositories and accession number(s) can be found below: https://www.ncbi.nlm.nih.gov/, accession ID: PRJNA784020.

## Ethics Statement

The animal study was reviewed and approved by the animal care protocol in this study followed commercial management practice and was approved by the Animal Welfare Committee of Liaocheng University. Written informed consent was obtained from the owners for the participation of their animals in this study.

## Author Contributions

YL conceived the study. YL and QM drafted the manuscript. YL, QM, ZZ, YZ, and MZ performed the experiments. GL and CW supervised the work and reviewed the manuscript. All authors contributed to the manuscript and approved the submitted version.

## Conflict of Interest

The authors declare that the research was conducted in the absence of any commercial or financial relationships that could be construed as a potential conflict of interest.

## Publisher’s Note

All claims expressed in this article are solely those of the authors and do not necessarily represent those of their affiliated organizations, or those of the publisher, the editors and the reviewers. Any product that may be evaluated in this article, or claim that may be made by its manufacturer, is not guaranteed or endorsed by the publisher.

## Footnotes

1
www.majorbio.com
