# Supplementary material for: Metabolic Alternations During Gestation in Dezhou Donkeys and the Link to the Gut Microbiota
Source: Front Microbiol. 2022 Mar 17;13:801976. doi: 10.3389/fmicb.2022.801976 (PMC8969422; doi:10.3389/fmicb.2022.801976)
Supplement: Supplementary file 1 [file Data_Sheet_1.doc]

Supplementary Materials

# Supplementary Figures and Tables

## Supplementary Figures


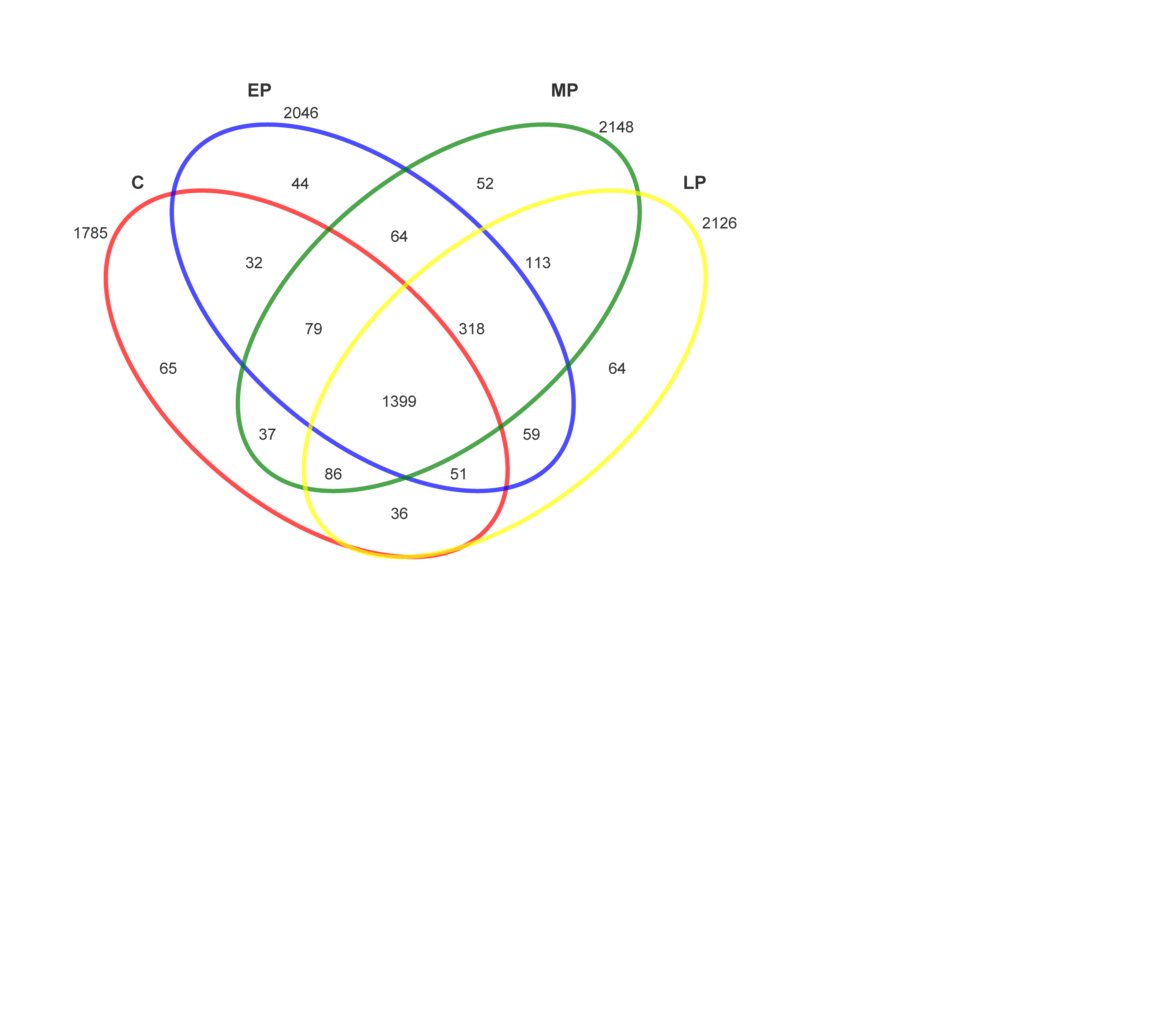


**Supplementary Figure 1.** A Venn diagram was generated to compare the OTUs among the different gestation stages. C, nonpregnancy as a control; EP, early-stage pregnancy; MP, middle-stage pregnancy; LP, late-stage pregnancy.


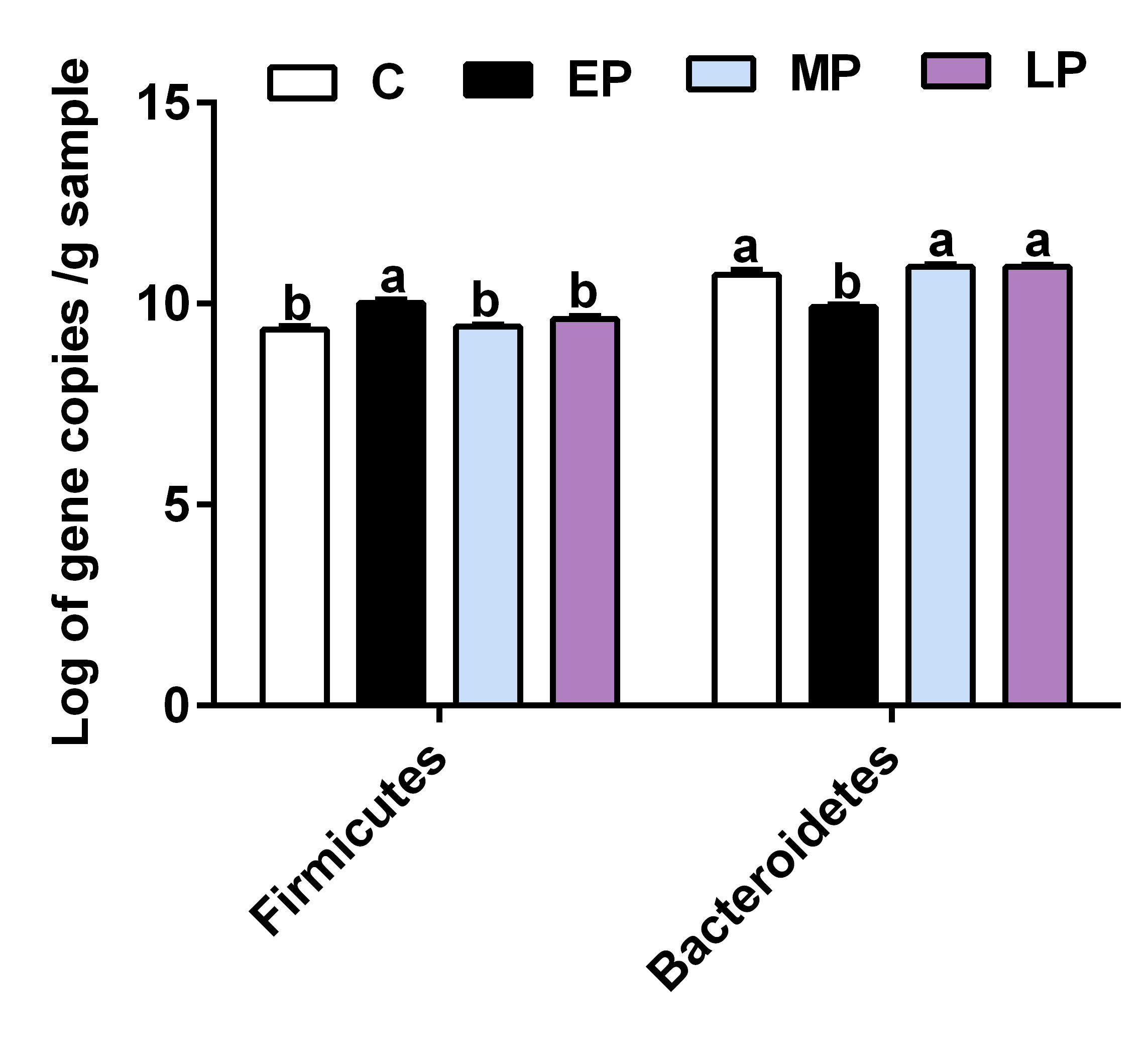


**Supplementary Figure 2.** The abundances of Firmicutes and Bacteroidetes in donkeys during different gestation stages. C, nonpregnancy as a control; EP, early-stage pregnancy; MP, middle-stage pregnancy; LP, late-stage pregnancy. Data indicate means ±SEM (n = 6). a,b,c Means with different letters are significantly different, p < 0.05.


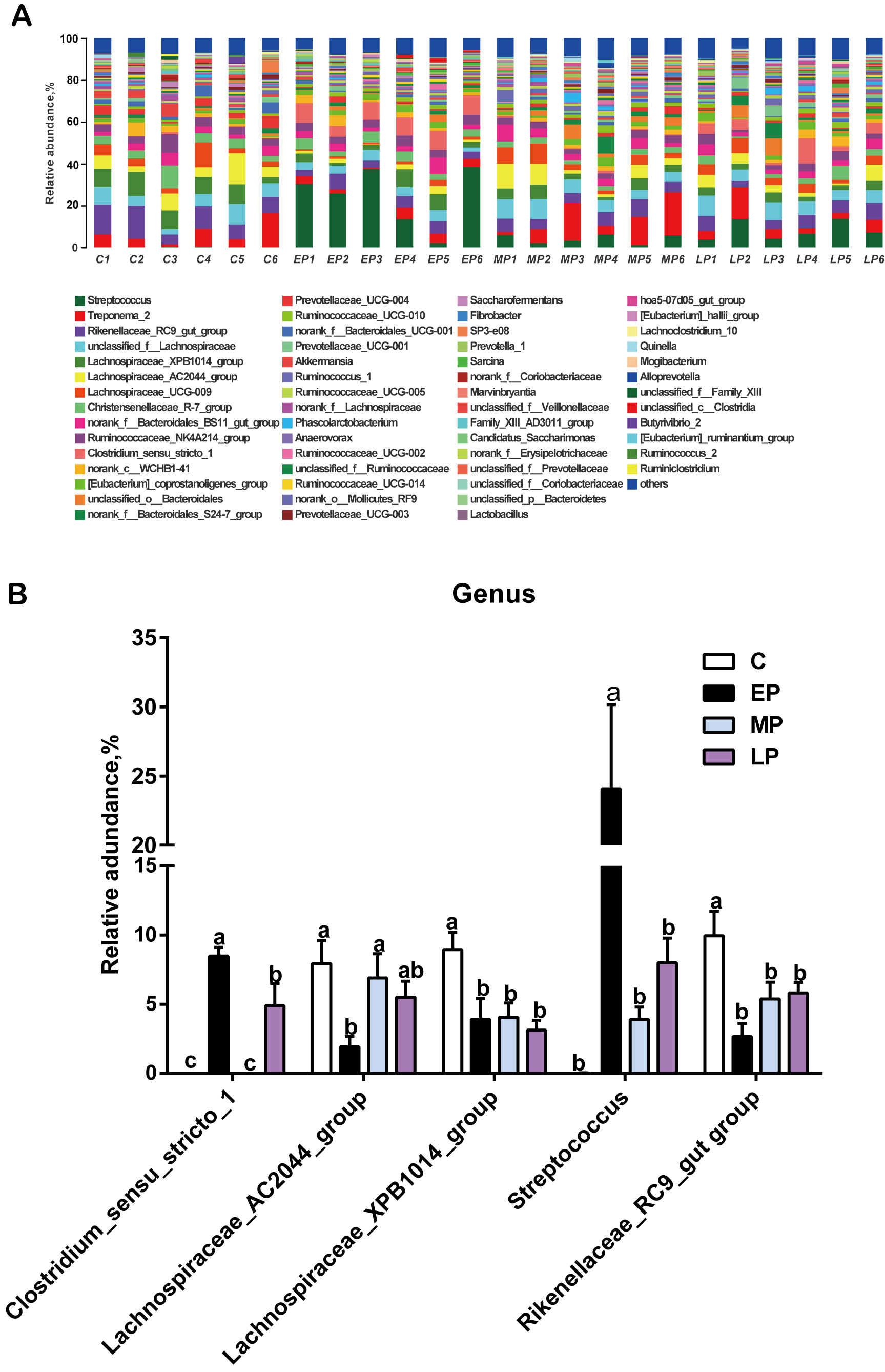


**Supplementary Figure 3.** The bacterial composition of the gut microbiota in donkeys during different gestation stages. (A) The gut bacterial composition at the genus level in donkeys during different gestation stages. (B) The relative abundances of genera *Clostridium_sensu_stricto_1*, *Lachnospiraceae_AC_2044_group*, *Lachnospiraceae_XPB1014_group*, *Streptococcus*, and *Rikenellaceae_RC9_gut_group* in donkeys during different gestation stages. C, nonpregnancy as a control; EP, early-stage pregnancy; MP, middle-stage pregnancy; LP, late-stage pregnancy. Data are expressed as means ± SEM. a,b,c Means with different letters are significantly different, p < 0.05.


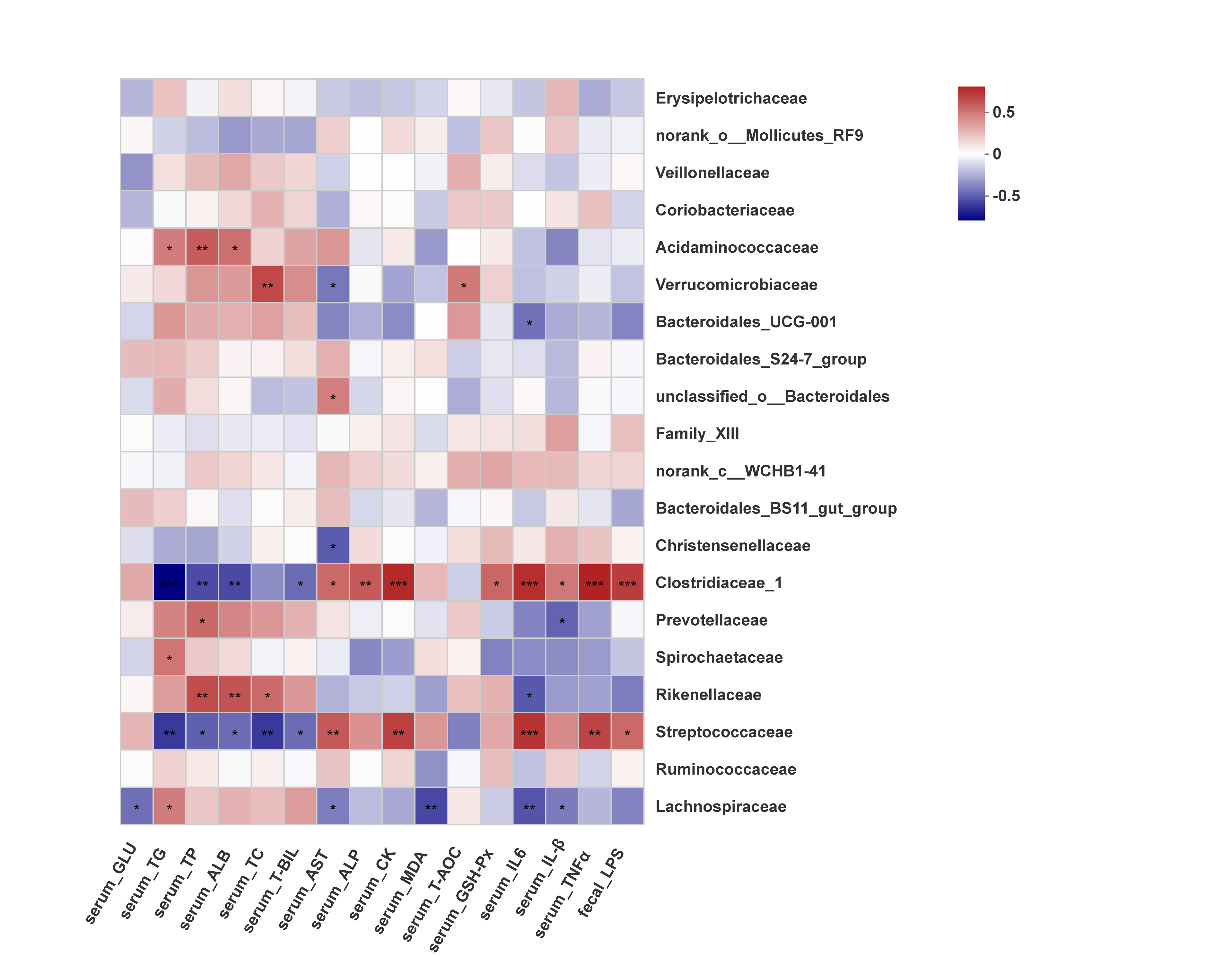


**Supplementary Figure 4.** Spearman correlation analysis. Heatmaps of Spearman correlation analysis between the abundances of bacterial families and biochemistry parameters related to metabolism and immune function.

## Supplementary Table

**Supplementary Table 1. Primers for amplifying target bacteria with qPCR**

| Target species | Primer sequence (5’-3’)^a^ | Product size (bp) | References |
| --- | --- | --- | --- |
| Firmicutes | F: CTGATGGAGCAACGCCGCGT  R: ACACYTAGYACTCATCGTTT | 448 | (Haakensen et al., 2008) |
| Bacteroidetes | F:GGARCATGTGGTTTAATTCGATGAT  R: AGCTGACGACAACCATGCAG | 127 | (Guo et al., 2008) |

Note: ^a^ Y = C or T.

**Supplementary materials references**

Guo, X., Xia, X., Tang, R., Zhou, J., Zhao, H., and Wang, K. (2008). Development of a real-time PCR method for Firmicutes and Bacteroidetes in faeces and its application to quantify intestinal population of obese and lean pigs. *Lett. Appl. Microbiol.* 47(5)**,** 367-373. doi: 10.1111/j.1472-765X.2008.02408.x.

Haakensen, M., Dobson, C.M., Deneer, H., and Ziola, B. (2008). Real-time PCR detection of bacteria belonging to the Firmicutes Phylum. *Int. J. Food Microbiol.* 125(3)**,** 236-241. doi: https://doi.org/10.1016/j.ijfoodmicro.2008.04.002.
